# Social-emotional problems among Swedish three-year-olds: an Item Response Theory analysis of the Ages and Stages Questionnaires: Social-Emotional

**DOI:** 10.1186/s12887-020-2000-y

**Published:** 2020-04-04

**Authors:** Masoud Vaezghasemi, Eva Eurenius, Anneli Ivarsson, Linda Richter Sundberg, Sven-Arne Silfverdal, Marie Lindkvist

**Affiliations:** 1 Department of Epidemiology and Global Health, Umeå University, SE-901 85 Umeå, Sweden; 2 Department of Clinical Science, Pediatrics, Umeå University, Umeå, Sweden

**Keywords:** Ages & Stages Questionnaires: Social-Emotional (ASQ:SE), Item Response Theory (IRT), Pre-school children, Social-emotional problems

## Abstract

**Background:**

There is enough evidence to believe that young children’s social-emotional problems can have a long-term effect if extra support is not given early. Therefore, early identification of such problems and any differences between boys and girls are of importance. We utilized the 36-month interval of the Ages and Stages Questionnaires: Social-Emotional (ASQ:SE) among 3-year-olds aiming: 1) to report the normative values of social-emotional problems for Swedish boys and girls; 2) to identify ASQ:SE items that are most commonly endorsed by children with high level of social-emotional problems (high score on ASQ:SE); 3) to assess whether certain ASQ:SE items differ between boys and girls at the same level of social-emotional problems; and 4) to examine whether ASQ:SE performs well in identifying children with high level of social-emotional problems (high score on ASQ:SE).

**Method:**

During 2014–2017, data were collected from 7179 three-year-old children (boys = 3719, girls = 3460) through Child Health Care in the Region Västerbotten in the northern part of Sweden. Unidimensionality was assessed by Confirmatory Factor Analysis and goodness-of-fit was reported. Item Response Theory was used to answer the aims of the study.

**Results:**

Items regarding interest in sexual words, too little sleep, disinterest in things around, unhappiness and self-injury were more commonly endorsed by children with high levels of social-emotional problems, as reported by their parents. For the same level of social-emotional problem, girls were more likely to demonstrate difficulties in occupying themselves, clinging behaviour and repetitive behaviour. On the other hand, boys were more likely to score high in items regarding destruction of things on purpose, difficulty to name friends and to express feelings. We have also found that the ASQ:SE is suitable for identifying children with high level of social-emotional problems.

**Conclusion:**

The salient point of our study was to increase knowledge about Swedish children’s social-emotional problems at 3-years of age based on the psychometric characteristics of the ASQ:SE using Item Response Theory model. The gender differences as well as those items that occurred at high levels of social-emotional problems should be of concern for everyday practice in Child Health Care.

## Introduction

Children’s ability to regulate their emotions and skilfully manage social interactions is critical to their development and well-being. Evidence indicate that young children’s social-emotional problems can have a long-term effect on their health if not identified and addressed early [1–5]. It has been demonstrated that 18% of children will meet the criteria for mental health problems by the age of 1.5 years by using a wide range of instruments and interviews [6]. These results point to the potential for mental health screening and intervention in the existing child health surveillance. Gender differences have been identified in children’s social and behavioural problems. Pre-school aged boys have significantly more externalizing behaviour, depression and developmental disorders compared to girls [7–10]. Early childhood is an important period for measuring and detecting mental health problems, and use of a screening instrument can aid early detection of children’s behavioural delays or disorders in order to enable early interventions suitable for both boys and girls.

The Ages and Stages Questionnaires: Social-Emotional (ASQ:SE) was developed in 2002 to reflect social and emotional problems in young children and has been widely used in the USA and internationally [11–13]. It consists of eight questionnaires (i.e. 6, 12, 18, 24, 30, 36, 48, and 60 months) with parental-reported social-emotional problems among children in seven areas; self-regulation, compliance, communication, adoptive functioning, autonomy, affect, and interaction with people. The second edition, published in 2015 (ASQ:SE-2) added a 2-month-old interval and expanded the age for children from 1 month to 72 months. In addition, several new items were added to evaluate early social-communication, adaptive, and autonomous behaviours [12–14]. Prior studies have evaluated the cost-effectiveness and examined the adequate psychometric properties of the instrument with promising results [11, 12].

Despite the growing concern about the increasing mental health problem in Sweden especially among children and young adults [15–17], our knowledge is very limited when it comes to pre-school children. Previous analyses, mainly in regard to social-emotional problems, focused on the psychometric properties of the instrument and provided a descriptive analysis of the ASQ:SE total score [10, 18, 19]. These studies have been valuable and have provided a basis for our understanding. However, further analysis is needed to deepen our knowledge concerning young children’s social and emotional functioning by examining the psychometric characteristics of the instrument itself. Therefore, we utilized the 36-month interval of the ASQ:SE among 3-year-olds aiming: 1) to report the normative values of social-emotional problems for Swedish boys and girls as reported by their parents; 2) to identify ASQ:SE items that are most commonly endorsed by children with high level of social-emotional problems (high score on ASQ:SE); 3) to assess whether certain ASQ:SE items differ between boys and girls at the same level of social-emotional problems; and 4) to examine whether ASQ:SE performs well in identifying children with high level of social-emotional problems (high score on ASQ:SE).

## Method

### Study setting

This study took place in the Region Västerbotten in the northern part of Sweden with 40 Child Health Care (CHC) centres for children up to the age of six years. Almost every child (about 3000 children annually) turning three years of age visits the CHC. The ASQ:SE 36-month interval was used for data collection within CHC at the 3-year-olds’ ordinary visit. Parents filled out the questionnaire at home prior to the visit and had a dialogue with the CHC nurse about their child on the basis of their responses during the visit. This data collection was done in collaboration with the Salut Child Health Promotion Programme [20].

### Study participants

Parental-reported data on 8214 3-year-olds were collected during January 2014–September 2017, which corresponds to a response rate of 80% of those attending CHC (that has an almost 100% coverage). Out of these, 7179 of the children remained for the study after considering the exclusion criteria. The fact that 1035 children were excluded was due to any of these three reasons: the parents did not consent to the research (*n* = 447); the age of the child could not be determined or was outside the recommended age range of the 36-month interval of the ASQ:SE (*n* = 513); and the number of unanswered ASQ:SE questions were more than three (*n* = 75).

### The instrument

We used the first edition of ASQ:SE (36-month interval) [13], which has been translated to Swedish according to established recommendations [21]. The allowed age ranged from 33 months and 0 days to 41 months and 29 days according to the ASQ:SE User’s Guide [22]. Child age was calculated by using the date for the check-up visit for the child at the CHC centre and the birth date of the child. The 36-month interval of the ASQ:SE consisted of 31 items, using a 3-point Likert scale (0, 5 or 10 points), and in addition, for each item the parents could indicate if it was a ‘concern’ for them (5 points). Thus, the range of possible total score was 0–465 (zero means no behaviour problem in any item and 465 means having problem in all items plus that parents expressed concern). In addition, the ASQ:SE includes three open-ended questions that should not be used for calculating the total score and were therefore not included.

### Data analysis and statistical considerations

#### Normative values

We started the analysis by examining ASQ:SE total scores based on the original ASQ:SE scoring to report normative values. Descriptive results were presented as numbers (n), mean, Standard Deviations (SD), medians, ranges, percentage (%) and percentiles. We calculated the total score and used the American cut-off value of 59 points to detect children with social-emotional problems according to the instructions in the ASQ:SE User’s Guide [22].

#### Social-emotional trait investigated by item response theory

We used Item Response Theory (IRT) to identify items that characterized children with high level of social-emotional problems [23]. It follows the measurement theory that aims to describe the relationship between an individual’s ability/vulnerability and the characteristics of the items across the continuum of the targeted latent trait [24]. The individual’s ability/vulnerability is defined by the latent trait. The latent trait is a continuous unidimensional construct and individuals with higher values on the latent trait have greater probability to endorse an item. In our study, the latent trait was social-emotional problems. Regarding the IRT analyses, the response option ‘concern’ was not included, because it is not part of the Likert scoring scale that evaluates the frequency of different child behaviours. All items where dichotomized to ‘no problem’ for those who scored 0 on any item whether it is always/often for “positive questions” (i.e. dose your child look at you when you are talking to him/her?) or it is seldom/never for “negative questions” (i.e. does your child cling to you more than you expect?), or ‘with problem’ for those who scored 5 and 10. This resulted in 31 binary items and all IRT analyses are based on this dichotomized scoring of items.

#### Internal consistency and unidimensionality

Cronbach’s alpha was used to assess the internal consistency of the items. In addition, model fit and unidimensionality were evaluated by Confirmatory Factor Analysis. This was done to reflect the relations of children’s social-emotional problems and the characteristics of items, and to ensure that items in ASQ:SE are targeting the same latent trait.

#### Item characteristic curve and threshold parameters

The relationship between the characteristics of each item and the latent trait was defined by the Item Characteristic Curve (ICC). This curve shows the probability of each ASQ:SE binary item to be endorsed as a function of the latent trait (social-emotional problem). The one-parameter IRT model is defined by the item threshold parameter, which is the point on the latent trait where there is 50% probability of the item being endorsed. Higher values on the threshold parameter indicate that only individuals with high levels on the latent trait score high on that item.

#### Differential item functioning

Differential Item Functioning (DIF) was used to determine whether an item exhibits uniformity between boys and girls across all the values of the social-emotional trait. DIF calculates the Mantel-Haenszel (MH) chi-square common Odds Ratio (OR) for dichotomously scored items. The MH statistics are used to determine whether an item favours one group relative to the other for all values of the latent trait. For instance, a common OR greater than 1 indicates DIF in favour of the focal group, which are girls in this analysis.

#### Test information function

The Test Information Function (TIF) is the sum of the ICCs for each value on the latent trait and TIF gives the test information for the entire questionnaire at each level of the latent trait. In other words, it basically tells how well the test is doing in estimating problem over the whole range of problem scores. The Standard Error of Measurement (SEM) at each level indicates the extent of measure preciseness, meaning lower SEM indicates higher precision or information.

All analyses were performed using STATA/SE version 16.0 (StataCorp, College Station, Texas 77,845 US).

### Ethics

The Regional Ethical Review Board in Umeå has approved the study (2013–268-31Ö). Only children whose parents have given written informed consent were included in the study. The study was carried out according to the ethical principles available in the Helsinki Declaration of 1975 (as revised in 1983).

## Results

### Normative values

We have included ASQ:SE responses for 7179 3-year old children with total scores ranging 0–215. Internal consistency measured with Cronbach’s alpha was 0.78. Generally, boys had higher scores (mean = 31.2, SD = 24.9) than girls (mean = 23.9, SD = 20.6). Further, ASQ:SE scores were higher for boys compared to girls across all the quartiles (boys with quartile 1 = 15, median = 25, quartile 3 = 45, and girls with quartile 1 = 10, median = 20, quartile 3 = 35) (Table 1). These results show that normative values for boys were nearer to the cut-off (59) for social-emotional problems than for girls.

**Table 1:** Normative values of the first edition of the Ages and Stages Questionnaires: Social-Emotional (ASQ:SE) for 36-month interval based on total scores for 7179 three-year-olds

ASQ:SE total score	Total sample ***N*** = 7179	Boys ***N*** = 3719	Girls ***N*** = 3460
Mean (SD)	27.7 (23.2)	31.2 (24.9)	23.9 (20.6)
Median (range)	25 (0–215)	25 (0–215)	20 (0–210)
Above the cut-off 59 (%)	9.1	12.3	5.6
1st Percentile	0	0	0
5th Percentile	0	0	0
10th Percentile	5	5	5
25th Percentile	10	15	10
50th Percentile	25	25	20
75th Percentile	40	45	35
90th Percentile	55	60	50
95th Percentile	70	75	60
99th Percentile	108	118	93

### Social-emotional trait investigated by IRT

#### Unidimensionality and model fit

Internal consistency of all the dichotomized items based on Cronbach’s alpha was 0.75. In addition, Confirmatory Factor Analysis was used to test for unidimensionality. Since normality could not be assumed, the method of quasi maximum likelihood estimation with robust standard error was used. This technique requires fewer assumptions than most other techniques. In particular, it merely assumes that the errors are independently distributed across observations and thus allows the errors to be heteroskedastic. The result was a Root Mean Square Error of Approximation (RMSEA) equal to 0.055 and a Coefficient of Determination (CD) equal to 0.83. The RMSEA is a good indicator if it is less than 0.08 and CD showed that 83% of the variance is explained by this model.

#### Item characteristic curve and threshold parameters

**Fig. 1:**
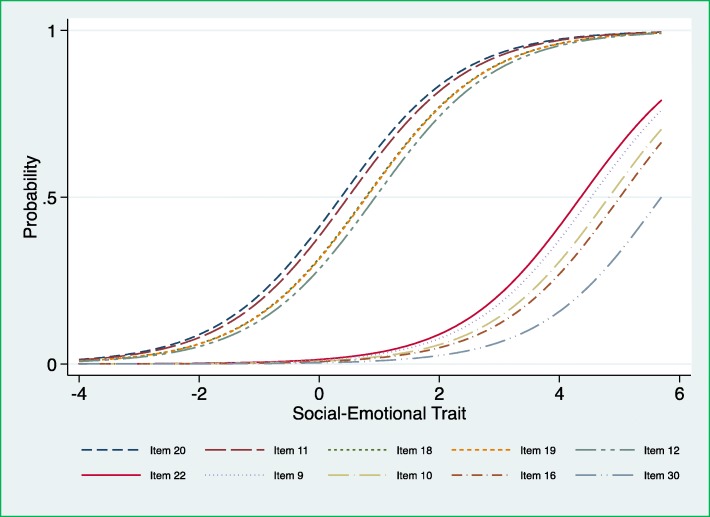
The probability of endorsing the five items that had the lowest and highest threshold parameter on the latent trait, respectively, using the first edition of the Ages and Stages Questionnaires: Social-Emotional (ASQ:SE) for 36-month interval

The threshold parameters are estimated from the ICC for each item (Fig. 1). The probability to endorse an item increases as the latent trait increases, i.e. social-emotional problems. The threshold parameter is the value on the latent trait where there is 50% probability that the item is endorsed. All the items were ranked based on their threshold parameter.

Figure 1 shows the first five and the last five items across the latent trait. For instance, the first five items (20, 11, 18, 19 and 12), and had the lowest threshold parameter on the latent trait, which means that those items were endorsed for most children with low level of social-emotional problems. However, the last five items (22, 9, 10, 16 and 30) had the highest threshold parameter on the latent trait, which means that those items were endorsed only for children with high levels of social-emotional problems. Table 2 presents a detailed information about these ten items including the number and percentages of children who had difficulties on these items. More information is available in Appendix in Table 3 where all the items are ranked based on their threshold parameters.

**Table 2:** Details of items endorsed at a very low and very high level of social-emotional problems derived from the first edition of the Ages and Stages Questionnaires: Social-Emotional (ASQ:SE) for 36-month interval

Items	Questions	Threshold parameter	N (%) of children with difficulties
*Five first items endorsed at a low level of social-emotional problems*			
20	Does your child check that you are nearby when he/she is exploring new places such as parks or a friend’s home?	0.36	3038 (42.6)
11	Does your child do what you ask him/her?	0.48	2866 (40.2)
18	Does your child comply with requests in everyday routines? For example, coming to the dinner table or putting away toys when you tell him/her.	0.77	2455 (34.3)
19	Does your child cry, scream or have fits of rage that are prolonged?	0.79	2455 (34.3)
12	Does your child seem to be more active than other children of the same age?	0.93	2248 (31.6)
*Five last items endorsed at a high level of social-emotional problems*			
22	Does your child hurt her/himself on purpose?	4.36	149 (2.1)
9	Does your child seem satisfied and happy?	4.53	126 (1.8)
10	Is your child interested in things around him/her? For example, people, toys and food?	4.82	95 (1.3)
16	Does your child sleep at least 8 h in 24 h?	5.01	80 (1.1)
30	Does your child show an unusual interest in, or knowledge of, sexual words/activities?	5.70	41 (0.6)

All items where dichotomized to ‘no problem’ for those who scored 0 on any item whether it is always/often for “positive questions” (e.g. Does your child seem satisfied and happy?) or it is seldom/never for “negative questions” (e.g. Does your child hurt her/himself on purpose?), or ‘with problem’ for those who scored otherwise

#### Differential item functioning

**Fig. 2:**
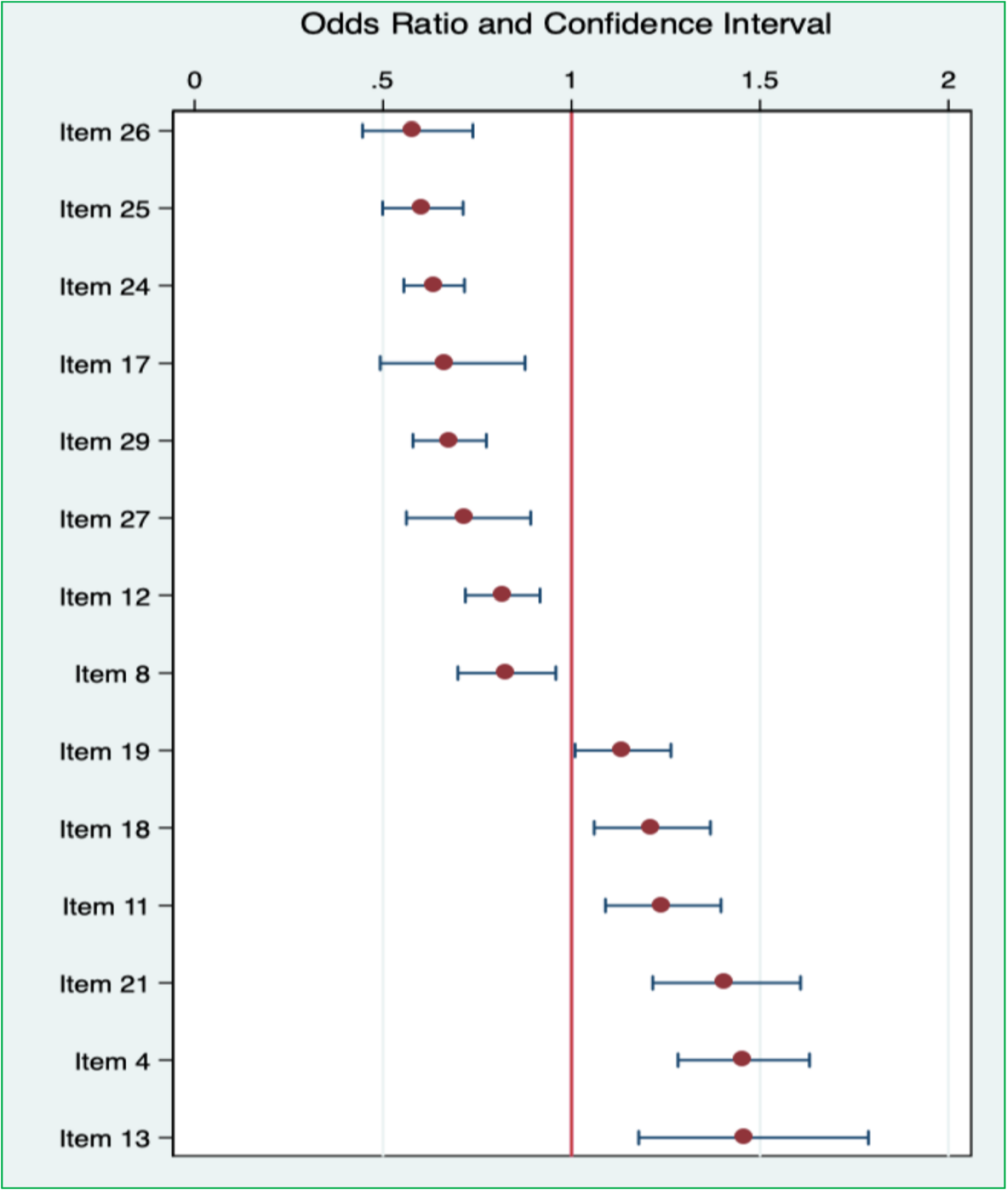
Mantel-Haenszel (MH) Odds Ratios for dichotomized items of the first edition of the Ages and Stages Questionnaires: Social-Emotional (ASQ:SE) for 36-month interval indicating gender differences at the same level of social-emotional trait with boys as reference category

The results from the DIF analysis showed that the parental reports of boys and girls were significantly different in almost half of the ASQ:SE items at the same level of social-emotional problems (Fig. 2). Boys are the reference group meaning that an OR greater than one indicates that DIF is in favour of the focal group or girls. Accordingly, the results suggest that when boys and girls had the same level of social-emotional problems, boys were more likely to exhibit difficulties in the following items ranked based on ORs: item *26 - Can your child name a friend? (OR = 0.57, CI = 0.44–0.74); item 25 - Does your child use words to describe his or her own and others’ feelings? For example, “I am happy”, “I don’t like it” or “She is sad” (OR = 0.59, CI = 0.50–0.71);* item *24 - Does your child break or destroy things on purpose? (OR = 0.63, CI = 0.55–0.71);* item *17 - Does your child use words to tell you what he/she wants or needs? (OR = 0.66, CI = 0.49–0.88)*; item *29 - Does your child try to hurt other children, adults or animals (*e.g. *by kicking or biting)? (OR = 0.67, CI = 0.58–0.77); item 27 - Do other kids like to play with your child? (OR = 0.71, CI = 0.56–0.89); item 12 - Does your child seem to be more active than other children of the same age? (OR = 0.81, CI = 0.72–0.92)*; and item *8 - Can your child switch activity without great difficulty?* E.g. *from playing to meals (OR = 0.82, CI = 0.70–0.96).*

Girls were more likely to exhibit difficulties in the following items ranked based on ORs: item *19 - Does your child cry, scream or have fits of rage that are prolonged? (OR = 1.13, CI = 1.01–1.26); item 18 - Does your child comply with requests in everyday routines? For example, coming to the dinner table or putting away toys when you tell him/her? (OR = 1.20, CI = 1.06–1.37)*; item *11 - Does your child do what you ask him/her? (OR = 1.23, CI = 1.09–1.40)*; item *21 - Does your child do things over and over again and get upset if you try to stop him/her? For example, rocking, flapping their hands, spinning round? (OR = 1.40, CI = 1.21–1.61)*; item *4 - Does your child cling to you more than you expect? (OR = 1.54, CI = 1.18–1.63);* and item *13 - Can your child occupy herself/himself for at least 5 min with things he/she enjoys (not including TV-watching)? (OR = 1.45, CI = 1.18–1.79).*

#### Test information function

**Fig. 3:**
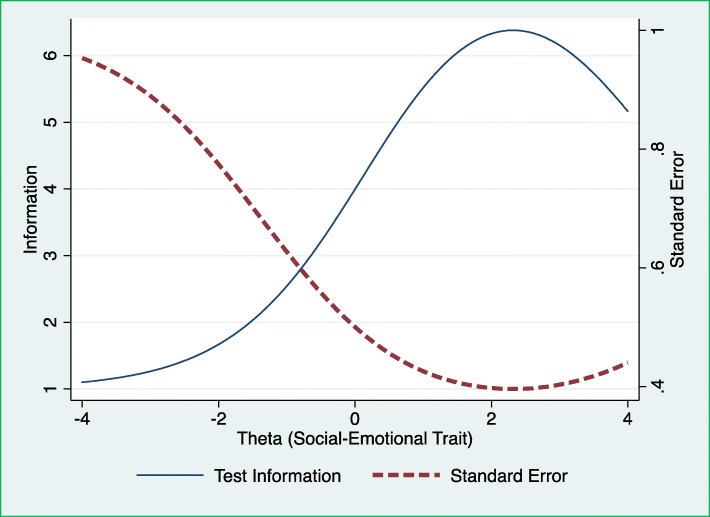
The Test Information Function (TIF) to explore the amount of the information from all items in the first edition of the Ages and Stages Questionnaires: Social-Emotional (ASQ:SE) for 36-month interval, across the social-emotional trait

Finally, by plotting TIF and SEM for each level of the latent trait (Fig. 3**)** we analysed at which levels of the latent trait/social-emotional problems the ASQ:SE works best and provides most information. In Fig. 3, the scale of TIF was set on the left and plotted by solid line. The scale of SEM was set on the right and plotted by dash line. Higher item information represents a lower SEM and higher reliability.

## Discussion

The main findings in our study were: 1) ASQ:SE scores were higher for boys compared to girls across all the quartiles (boys with quartile 1 = 15, median = 25, quartile 3 = 45 and girls with quartile 1 = 10, median = 20, quartile 3 = 35), which means that normative values for boys were nearer to the cut-off (59) for social-emotional problems; 2) The five items with highest threshold parameter were more commonly endorsed by children with high level of social-emotional problems. These items were about unusual interest in sexual words/actions, too little sleep, disinterest in things around, unhappiness and self-injury; 3) At the same level of social-emotional problems, girls demonstrated difficulties in occupying themselves, clinging behaviour and repetitive behaviour, while boys’ social-emotional problems more often comprised difficulty to name friends, difficulty to express feelings and destruction of things on purpose; 4) ASQ:SE performed very well in identifying children with high level of social-emotional problems.

### Items that contributed to high levels of social-emotional problems

Previous study on the distribution of the ASQ:SE items [10] has shown that parents were mainly concerned about the following items: children’s eating situation, problems when asked whether the child checks that you are nearby, does what you ask him/her, complies with your requests, cries or screams for a long time, or about a child’s hyperactivity. Although many children may have difficulties in these areas, it does not necessarily mean that they should be considered having social-emotional problems. According to our present study all these items occurred at the level of very low social-emotional trait/problems. In contrast, very few children demonstrated – based on parental-report – problems on items regarding interest in sexual words and activities, too little sleep, disinterest in things around, unhappiness, and self-injury. We have shown that these behaviours are more commonly exhibited by children with high levels of social-emotional problems. Thus, we suggest that children with these latter behaviours are given attention at times when the complete ASQ:SE is inaccessible or has not been carried out, as is still the situation in most other CHC settings in Sweden and elsewhere.

### Gender differences in ASQ:SE total score and items

Gender differences in emotional and behavioural problems have previously been observed among pre-schoolers in Sweden [7] and outside Scandinavia [25, 26]. We showed that a higher percentage of boys (12.3%) had a parental reported score above the cut-off compared to girls (5.6%). Other authors have reported that, among children of school age the gender imbalance is reversed [15]. One reason for such discrepancy may be that, at an early age children’s emotional and behavioural problems are mainly assessed through teachers and parental observations and questionnaires, while older children usually self-report their problems. Furthermore, the gender difference in ASQ:SE scores has been acknowledged in the ASQ:SE-2 User’s Guide when it comes to referral [14]. It is suggested that girls should be referred for specialized assessment and earlier therapeutic interventions already with scores lower than the cut-off point (i.e. in the monitoring zone). The gender differences shown through our IRT analysis suggest that at the same level of social-emotional problems both boys and girls exhibited difficulties in some areas of self-regulation. However, boys were more likely to have interaction and communication problems, while girls were more likely to show vulnerabilities in autonomy and compliance. Our results are in line with a previous study on ASQ:SE conducted on a population sample from Brazil, China, South Korea and USA [27]. Their results indicated that with equal levels of social-emotional problems, parents of girls were more likely to report internalizing problematic behaviours than parents of boys, such as ‘*Does your child cling to you more than you expect?’*. On the other hand, parents of boys were more likely to report externalizing problematic behaviours, such as ‘*Does your child try to hurt other children, adults, or animals?*’. It has been shown that, boys’ expressions of mental health problems may be easier to observe as these are more externalized and therefore largely reported by parents, while internalized psychological symptoms are more common among girls [15]. The latter demand more developed communication skills to be verbalized, and thus could easily be missed in reports by parents of younger children. However, whether the gender differences detected in our analysis are because of the children’s own tangible performance or their parents’ perceptions and expectations remains unclear and requires further investigations.

### Strengths and weaknesses of the study

One of the important strengths of this study is that the results are derived from a comprehensive population-based study with high participation rates (about 80%). The use of ASQ:SE is another asset of our study, as it has shown to be a promising tool in detecting social-emotional problems in pre-school children [13]. Applying advanced statistical methods such as IRT is another advantage of our study, as it has made it possible to examine the quality of each item and understand how each item functions across the spectrum of the latent trait of the social-emotional problems.

There are also some weaknesses in our study that need to be acknowledged, however, these does not challenge our conclusions. The cut-off point of 59 was based on an American study [22] and may not entirely reflect the social-emotional problems in our population. However, using this cut-off made it possible to compare our results with many other international studies, which has been valuable. We have used only the Swedish and English version of the ASQ:SE. This likely contributes to lower response rate among immigrant families who might have different cultural beliefs and perceptions in regard to children’s socio-emotional behaviours. Although our aim was not to investigate the difference across cultures or ethnicities, the gender differences detected in our analysis were in line with other cross-cultural studies [27].

## Conclusion

The salient point of our study was to increase knowledge about Swedish children’s social-emotional problems at 3 years of age based on the psychometric characteristics of the ASQ:SE using the IRT model. Children with high level of social-emotional problems were rated high on items regarding interest in sexual words and activities, too little sleep, disinterest in things around, unhappiness, and self-injury. Boys had higher probability of difficulties in social interactions and externalizing behaviours, while girls were more likely to demonstrate internalizing problematic behaviours. These gender differences as well as those items that occurred at high levels of social-emotional problems should be of concern for everyday practice in Child Health Care.

## Data Availability

Region Västerbotten originally collected the data for a child health survey (https://www.regionvasterbotten.se/salut). We accessed data for the present study after approval from both the Region Västerbotten and the Ethical Vetting Board. The data are not publicly available but access for replication analyses is possible.

## Appendix

**Table 3:** Estimation of threshold parameters for all ASQ:SE^a^ binary items as a function of the social emotional trait among three-year-old boys (*n* = 3719) and girls (*n* = 3460). Ranked from lowest to highest

ASQ:SE^a^ items	Threshold parameter	95% Confidence Interval
Discrimination	0.99	(0.96–1.01)
20	0.36	(0.31–0.42)
11	0.48	(0.42–0.54)
18	0.77	(0.71–0.84)
19	0.79	(0.73–0.85)
12	0.93	(0.87–1.00)
7	1.02	(0.95–1.08)
14	1.08	(1.02–1.15)
4	1.13	(1.06–1.19)
25	1.21	(1.14–1.27)
6	1.67	(1.59–1.74)
21	1.76	(1.68–1.84)
29	1.79	(1.71–1.87)
8	1.92	(1.84–2.00)
23	2.33	(2.24–2.43)
25	2.43	(2.33–2.53)
15	2.43	(2.34–2.53)
2	2.53	(2.43–2.63)
28	2.81	(2.70–2.92)
5	2.86	(2.75–2.98)
13	2.92	(2.80–3.03)
27	3.07	(2.95–3.19)
1	3.25	(3.12–3.37)
26	3.31	(3.17–3.44)
31	3.45	(3.31–3.59)
17	3.59	(3.44–3.73)
3	3.94	(3.78–4.11)
22	4.36	(4.16–4.55)
9	4.53	(4.32–4.74)
10	4.82	(4.59–5.06)
16	5.01	(4.75–5.26)
30	5.7	(5.36–6.04)

^a^The first edition of the Ages and Stages Questionnaires: Social-Emotional for 36-month interval

## Abbreviations

ASQ:SE: Ages & Stages Questionnaires: Social-Emotional
CD: Coefficient of Determination
CHC: Child Health Care
DIF: Differential Item Functioning
ICC: Item Characteristic Curve
IRT analysis: Item Response Theory analysis
MH: Mantel-Haenszel
OR: Odds Ratio
RMSEA: Root Mean Square Error of Approximation
SD: Standard Deviation
SEM: Standard Error of Measurement
TIF: Test Information Function
